# Influenza A-triggered Bickerstaff brainstem encephalitis successfully treated with therapeutic plasma exchange: A case report

**DOI:** 10.1016/j.htct.2025.103843

**Published:** 2025-05-10

**Authors:** Piotr F. Czempik, Małgorzata Pięta, Tomasz Jaworski, Piotr Liberski

**Affiliations:** a Faculty of Medical Sciences in Katowice, Medical University of Silesia, Katowice, Poland; b University Clinical Center of Medical University of Silesia, Katowice, Poland

## Introduction

Bickerstaff brainstem encephalitis (BBE) is an extremely rare autoimmune disease with the first reports being published in the 1950s [1]. The most characteristic neurological symptoms of BBE include the following: ophthalmoplegia, ataxia, impaired consciousness, limb weakness, facial paralysis, positive Babinski’s sign and impaired superficial sensation [2]. Impaired consciousness was shown to be most likely caused by dysfunction of the ascending reticular activating system [3]. Diagnosis of the disease is complex due to similarity of symptoms to other neurologic diseases (e.g. meningitis, encephalitis). Initially, the diagnosis in our case was infective meningitis or encephalitis, but it was revised to BBE after ruling out neuro-infection. Laboratory confirmation of BBE is often delayed as in most institutions anti-ganglioside antibody (anti-GQ1b) tests are performed externally, leading to prolonged turn-around time. There are three diseases in which anti-ganglioside antibodies are present – BBE, Miller Fisher syndrome (MFS), Guillaine-Barre syndrome (GBS) – and overlapping of diseases may occur. Characteristic for MFS are ophthalmoplegia, ataxia, areflexia, and obtunded consciousness. Some authors view BBE and MFS as a continuous spectrum of one disease, the so-called Fisher-Bickerstaff syndrome [4,5]. Anti-ganglioside antibodies, produced in response to infective agents (mostly bacteria) sharing ganglioside structures, can damage myelin sheaths through molecular mimicry [6].

## Case presentation

A 32-year-old patient was admitted to the local intensive care unit (ICU) due to deteriorating consciousness with a preliminary diagnosis of neuro-infection or autoimmune encephalitis. Past medical history was negative. The current presentation was preceded by contact with an influenza A-positive child. Three days before hospital admission, the patient became feverish (up to 40 °C), complained of nasal congestion and dry cough. Notably, the patient reported numbness of hands and feet that started one day before admission. On the day of hospital admission, the patient reported spinning dizziness intensifying on standing and sitting and with head movements, and nausea. On admission to the neurology department the patient was conscious, with logical verbal contact, oriented, with no meningeal symptoms, the only abnormality in the neurologic examination was first degree fine-wave nystagmus when looking to the right. An antigen test for respiratory viruses was negative, C-reactive protein was 22.3 mg/L, cerebrospinal fluid (CSF) was colorless and clear, with elevated cytosis (17 cells/µL; norm <5 cells/µL) and slightly elevated glucose (78.3 mg/dL with 76 % of serum concentration; norm 40–60 mg/dL with 60 % of serum concentration) and immunoglobulin G concentration (4.1; norm 1–3 mg/dL). A non-contrast computed tomography (CT) of the head showed only paranasal sinusitis. A fast deterioration of the neurological status was noted – the patient became periodically uncooperative with dysarthric speech, positive Kernig's sign, ophthalmoplegia with predominant leftwards gaze, ataxia, vivid deep reflexes, and positive Babinski sign on the left side. First-line treatment included empirical broad-spectrum antibiotic therapy, anti-viral agents, and corticosteroids. Initial treatment was unsuccessful: the patient became more obtunded and bradypnoe was noticed, therefore the patient was transferred to the ICU where he was intubated and mechanical ventilation was commenced (<24 h after hospital admission). Appropriate samples were obtained for additional laboratory and microbiological tests. Further diagnostic imaging included: contrast-enhanced CT of the head, CT angiogram of the head, contrast-enhanced magnetic resonance imaging (MRI) and contrast-enhanced CT of the chest, abdomen and pelvis however, no abnormalities apart from paranasal sinusitis were detected. The electroencephalogram revealed disturbed spatial organization and generalized retardation of basic activity. A nerve conduction study (NCS) showed mostly axonal motor-sensory polyneuropathy with predilection to lower extremities. Following exclusion of neuro-infection, the constellation of the symptoms of external ophthalmoplegia, ataxia, and deterioration of consciousness, as well as the result of NCS, made the diagnosis of BBE most probable. The patient was scheduled for emergency therapeutic plasma exchange (TPE). A dialysis cannula was inserted through the right internal jugular vein and a series of five procedures scheduled every other day was carried out. A 5000 mL volume of 4 % human albumin solution was utilized as the substitution fluid. The procedure was performed using a standard continuous renal replacement therapy apparatus (multiFiltratePRO, Fresenius Medical Care, Germany) and a special filter (Plasma Flux P2 dry, Fresenius Medical Care, Germany). Standard therapeutic doses of heparin sodium were used for anticoagulation during the procedure. Following the initiation of TPE, a blood sample for anti-ganglioside antibodies (anti-GM1, anti-GD1b, anti-GQ1b) was sent for analysis but the results came back negative. During the course of TPE the patient showed multiple episodes of vegetative excitation with tachycardia, hypertension, sweating, and muscle tension. As the duration of mechanical ventilation was prolonged (>7 days), percutaneous dilatational tracheotomy (Griggs technique) was performed ^7^. Due to inability to feed the patient orally, a percutaneous gastrostomy was inserted. Three days after the last TPE procedure the patient regained consciousness and non-verbal logical contact. The only laboratory test that came back positive was anti-glutamic acid decarboxylase antibodies (a high titer >2000 IU/mL). The patient was then transferred to the neurology department and later to the neurological rehabilitation department where he made full neurological recovery.

## Discussion

Clinical features of our patient were characteristic of anti-GQ1b-positive BBE: prior upper respiratory tract infection, mildly elevated cell count and protein concentration in the CSF, normal brain MRI (performed twice: during diagnosis and after regaining consciousness), and relatively fast return of consciousness [8]. The negative anti-GQ1b test in this patient could be due to the fact that a blood sample was collected only after starting the patient on TPE, by which time pathologic antibodies could have been removed or their concentration significantly decreased. Nevertheless, initiation of appropriate therapy should not wait until these test results come back. As soon as neuro-infection was excluded by negative CSF cultures, TPE was started. In our institution standard CSF cultures (final result after approximately five days) are used to establish the etiologic agent in neuro-infections: this could be achieved in a matter of one hour if polymerase chain reaction was used. The examination of CSF in BBE may show increased or normal cytosis and elevated protein [9]. In our case both were elevated. A nerve conduction study in BBE may reveal axonal demyelination [10]. In this case, NCS also revealed axonal involvement. This test is particularly useful if there is a suspicion of BBE with overlapping GBS. In the case of severe or rapidly progressing BBE, the initiation of TPE seems to be the most beneficial therapeutic option, as intravenous immunoglobulins and corticosteroids may not be effective. Physicians performing TPE should be aware of this disease that can present in both children and adults, in order to commence treatment at an early stage of BBE.

BBE is a rare disease with partly unspecific neurological symptoms. Following exclusion of an infective cause of neurological symptoms, patients should optimally be started on TPE as alternative therapies may not be effective. The turn-around time for confirmatory laboratory tests is prolonged and should not delay the start of appropriate treatment.

## Contribution statement

**PFC:** Conceptualization. **PFC, MP, TJ, PL:** Manuscript Writing & Editing.

## Conflicts of interest

None.
